# Computational prediction of protein interactions related to the invasion of erythrocytes by malarial parasites

**DOI:** 10.1186/s12859-014-0393-z

**Published:** 2014-11-30

**Authors:** Xuewu Liu, Yuxiao Huang, Jiao Liang, Shuai Zhang, Yinghui Li, Jun Wang, Yan Shen, Zhikai Xu, Ya Zhao

**Affiliations:** Department of Pathogenic Biology, The Fourth Military Medical University, Xi’an, 710032 P. R. China

**Keywords:** *Plasmodium falciparum*, Expectation maximization, Fast Fourier transform, Invasion, Red blood cell

## Abstract

**Background:**

The invasion of red blood cells (RBCs) by malarial parasites is an essential step in the life cycle of *Plasmodium falciparum*. Human-parasite surface protein interactions play a critical role in this process. Although several interactions between human and parasite proteins have been discovered, the mechanism related to invasion remains poorly understood because numerous human-parasite protein interactions have not yet been identified. High-throughput screening experiments are not feasible for malarial parasites due to difficulty in expressing the parasite proteins. Here, we performed computational prediction of the PPIs involved in malaria parasite invasion to elucidate the mechanism by which invasion occurs.

**Results:**

In this study, an expectation maximization algorithm was used to estimate the probabilities of domain-domain interactions (DDIs). Estimates of DDI probabilities were then used to infer PPI probabilities. We found that our prediction performance was better than that based on the information of *D. melanogaster* alone when information related to the six species was used. Prediction performance was assessed using protein interaction data from *S. cerevisiae*, indicating that the predicted results were reliable. We then used the estimates of DDI probabilities to infer interactions between 490 parasite and 3,787 human membrane proteins. A small-scale dataset was used to illustrate the usability of our method in predicting interactions between human and parasite proteins. The positive predictive value (PPV) was lower than that observed in *S. cerevisiae*. We integrated gene expression data to improve prediction accuracy and to reduce false positives. We identified 80 membrane proteins highly expressed in the schizont stage by fast Fourier transform method. Approximately 221 erythrocyte membrane proteins were identified using published mass spectral datasets. A network consisting of 205 interactions was predicted. Results of network analysis suggest that SNARE proteins of parasites and APP of humans may function in the invasion of RBCs by parasites.

**Conclusions:**

We predicted a small-scale PPI network that may be involved in parasite invasion of RBCs by integrating DDI information and expression profiles. Experimental studies should be conducted to validate the predicted interactions. The predicted PPIs help elucidate the mechanism of parasite invasion and provide directions for future experimental investigations.

**Electronic supplementary material:**

The online version of this article (doi:10.1186/s12859-014-0393-z) contains supplementary material, which is available to authorized users.

## Background

The ability of a malaria parasite to invade red blood cells (RBCs) is a key step for its survival and pathogenesis. The malarial parasites can successfully invade RBCs by several mechanisms, including rhoptry and microneme protein secretion, tight junction formation, and acto-myosin motor assembly [1,2]. Previous investigations revealed that surface protein interactions between parasite and human surface proteins are necessary to allow parasites to invade RBCs. However, although several surface proteins were identified to be involved in this process [3-5], the molecular basis of invasion remains poorly understood. Indeed, specific interactions between parasite proteins and human proteins remain largely undiscovered. Elucidation of the molecular basis of such interactions is critical to develop new intervention strategies against malaria.

Although high-throughput biological experiments such as yeast two-hybridization and avidity-based extracellular interaction screen can be conducted to reveal interactions between parasite proteins and human proteins [6,7], there are several technical difficulties in conducting such experiments with malarial parasites. A major difficulty is in expression of parasite proteins in yeasts or mammalian cells because of high AT content (approximately 80%) in the genome of *P. falciparum* [8]. For this reason, large-scale experimental validation of protein interaction networks is often ruled out in the case of malaria parasite. Moreover, inherent shortcomings of high-throughput techniques, such as bias toward yeast homologs, large false positive rates, and low coverage, could contribute to the significant inaccuracies of projected interactions [9]. Computational methods have been developed and used to predict protein interactions when experimental methods are not feasible. For example, Date et al. utilized a Bayesian framework to integrate different data sources, including polygenetic profiles, gene expression profiles, and Rosetta stone fusion genes [10]. Positive and negative high confidence (HC) datasets derived from Gene Ontology and KEGG databases were used to determine the likelihood scores of each interaction. By setting a threshold, they generated a protein–protein interaction (PPI) network with sensitivity of about 21%. Based on the resulting network, several uncharacterized proteins were assigned to various biological processes. Another approach based on protein sequence similarity was developed and implemented to predict putative protein interactions between human and malaria parasite [11]. Candidate interactions were then assessed by random forest classification and further filtered in terms of expression and molecular characteristics. The resulting network revealed that parasites possibly utilize their proteins in a combined manner by predominantly targeting hub proteins. Although several predicted protein networks have been constructed, predictions of membrane protein interactions related to parasite invasion have not been conducted before.

In this study, membrane protein interactions between human and *P. falciparum* were predicted to elucidate the protein interactions involved in parasite invasion of RBCs. Considering that a protein domain serves as a unit of protein-protein interactions and is evolutionally conserved, a model was developed to relate protein interaction probabilities with domain interaction probabilities. In the present study, an expectation maximization (EM) algorithm proposed by Liu et al. was used to estimate the probabilities of domain-domain interactions (DDIs) [12]. The EM algorithm employs a likelihood-based approach and exhibits good performance in estimating DDI probabilities [13]. In this approach, PPIs and DDIs were treated as random variables. The probabilities of DDIs were computed on the basis of information of PPIs after false positive rate (*f*_*p*_) and false negative rate (*f*_*n*_) were specified. DDI estimates were then used to infer plausible interactions between human and parasite membrane proteins.

## Methods

### Collection of physical protein-protein interaction data

The protein**-**domain relationships of each protein in six species, including *Arabidopsis thaliana*, *Caenorhabditis elegans*, *Drosophila melanogaster*, *Homo sapiens*, *Mus musculus*, and *Schizosaccharomyces pombe*, were extracted from the PFAM database [14]. To reduce the false positive rate (FPR) of protein**-**domain relations, we defined a significant protein**-**domain relationship (PDR) as one with E-value less than 1E-5 for proteins in *A. thaliana*, *C. elegans*, *D. melanogaster*, *H. sapiens*, *M. musculus*, and less than 1E-4 for proteins in *S. pombe*. Proteins with significant PDRs were represented by their respective Entrez gene ID or gene symbol. The physical protein-protein interaction data of these six organisms were collected from the BioGRID database [15]. We only considered interactions among proteins with significant PDRs. After removing redundant interactions, we obtained 9,478 protein interactions from *A. thaliana*, 3,052 interactions from *C. elegans*, 21,752 interactions from *D. melanogaster*, 94,396 interactions from *H. sapiens*, 7,409 interactions from *M. musculus*, and 3,828 interactions from *S. pombe*. Detailed information on the collected protein interactions for each species used in our study can be found in Additional file 1.

### High-confidence dataset preparation

Three high confidence datasets were constructed and used to evaluate the reliability of prediction. The first HC dataset contained DDIs collected from iPFAM and 3DID databases [16,17]. A total of 6,634 structurally verified physical domain interactions were found in these two databases. After removing domains not found in *D. melanogaster*, we constructed a DDI matrix containing information of interactions among 1,515 domains. The second high confidence dataset consisted of protein interactions from *S. cerevisiae*, in which physical protein interactions have been widely investigated. The PPIs in *S. cerevisiae* were also collected from BioGrid database. We defined PDR as significant when its E-value was less than 1E-4. After removing proteins containing domains not found in the six species studied here, we obtained 49,084 interactions among 3,960 proteins. We considered these 49,084 interactions as positive interactions and the remaining protein pairs were considered to be non-interacting (negative interactions). The last high confidence dataset was a small-scale dataset that contained 456 experimentally determined interactions between human and parasite proteins [18]. After removing proteins that do not satisfy the PDR condition, we obtained 132 interactions between 66 parasite proteins and 107 human proteins. The first and second datasets were used to evaluate the reliabilities of our prediction in DDIs and PPIs, respectively. The last dataset was utilized to assess the prediction performance of protein interactions between humans and parasites.

### EM algorithm

We estimated DDIs using the EM algorithm [12,19]. The interaction probability (*P*_*inter*_) between proteins *P*_*i*_ and *P*_*j*_ was expressed as follows:

$$ {\boldsymbol{P}}_{\boldsymbol{inter}}=1-{\displaystyle \prod_{{\boldsymbol{D}}_{\boldsymbol{mn}}^{\boldsymbol{ij}}\in {\boldsymbol{P}}_{\boldsymbol{ij}\boldsymbol{k}}}}\left(1-{\boldsymbol{\lambda}}_{\boldsymbol{mn}}\right) $$

where *P*_*ijk*_ represents the protein pair *P*_*i*_ and *P*_*j*_ in organism *k* (*k* = 1… 6) and *λ*_*mn*_ is the interaction probability of domain pair *D*_*m*_ and *D*_*n*_. We defined ${D}_{mn}^{ij}=1$ if *D*_*m*_ and *D*_*n*_ interacted in the protein pair *P*_*i*_ and *P*_*j*_ and ${D}_{mn}^{ij}\kern0.5em =\kern0.5em 0$ otherwise. ${D}_{mn}^{ij}\in {P}_{ijk}$ denotes all domain pairs from *P*_*i*_ and *P*_*j*_ in organism *k*. The probability of interaction between proteins *P*_*i*_ and *P*_*j*_ in the experiments was expressed as follows:

$$ {\boldsymbol{P}}_{\boldsymbol{observed}}=\left(1-{\boldsymbol{P}}_{\boldsymbol{inter}}\right){\boldsymbol{f}}_{\boldsymbol{p}}+{\boldsymbol{P}}_{\boldsymbol{inter}}\left(1-{\boldsymbol{f}}_{\boldsymbol{n}}\right) $$

where *f*_*p*_ and *f*_*n*_ represent the FPR and the FNR of protein interaction data, respectively. *f*_*p*_ was calculated using the formula below when *f*_*n*_ and the average number of interaction partners were designated:

$$ {\boldsymbol{f}}_{\boldsymbol{p}}=\frac{2{\boldsymbol{N}}_{\boldsymbol{o}}-\left(1-{\boldsymbol{f}}_{\boldsymbol{n}}\right)\boldsymbol{m}\boldsymbol{t}}{\left(\boldsymbol{m}+1\right)\boldsymbol{m}-\boldsymbol{m}\boldsymbol{t}} $$

where *m* and *t* represent the protein number and the average number of interacting partners, respectively, and *N*_*o*_ is the number of observed PPIs. We assumed that *f*_*p*_ and *f*_*n*_ are similar across the six species. The likelihood function characterizing the probability of the observed protein interaction data across six species was expressed as follows:

$$ \boldsymbol{L}={\displaystyle \prod }{\left({\boldsymbol{P}}_{\boldsymbol{observed}}\right)}^{{\boldsymbol{O}}_{\boldsymbol{ijk}}}*{\left(1-{\boldsymbol{P}}_{\boldsymbol{observed}}\right)}^{\left(1-{\boldsymbol{O}}_{\boldsymbol{ijk}}\right)} $$

If protein *P*_*i*_ was interacting with protein *P*_*j*_ in species *k*, *O*_*ijk*_ = 1; otherwise, *O*_*ijk*_ = 0. After specifying *f*_*n*_ and *f*_*p*_, we can estimate *λ*_*mn*_ using the EM algorithm. The EM algorithm consisted of E- and M-steps. In the E-step, ${D}_{mn}^{ij}$ expectation should be computed on the basis of the observed PPI data. For a specific ${\lambda}_{mn}^{\left(t-1\right)}$, we used the following equation:

$$ \boldsymbol{E}\left({\boldsymbol{D}}_{\boldsymbol{mn}}^{\boldsymbol{ij}}\Big|{\boldsymbol{\lambda}}_{\boldsymbol{mn}}^{\left(\boldsymbol{t}-1\right)}\right)=\frac{{\boldsymbol{\lambda}}_{\boldsymbol{mn}}^{\left(\boldsymbol{t}-1\right)}*{\left(1-{\boldsymbol{f}}_{\boldsymbol{n}}\right)}^{{\boldsymbol{O}}_{\boldsymbol{ij}\boldsymbol{k}}}*{\boldsymbol{f}}_{\boldsymbol{n}}^{\left(1-{\boldsymbol{O}}_{\boldsymbol{ij}\boldsymbol{k}}\right)}}{{\left({\boldsymbol{P}}_{\boldsymbol{observed}}\right)}^{{\boldsymbol{O}}_{\boldsymbol{ij}\boldsymbol{k}}}*{\left(1-{\boldsymbol{P}}_{\boldsymbol{observed}}\right)}^{\left(1-{\boldsymbol{O}}_{\boldsymbol{ij}\boldsymbol{k}}\right)}} $$

After obtaining the ${D}_{mn}^{ij}$ expectation, we updated ${\lambda}_{mn}^t$ in the M-step using the following equation:

$$ {\boldsymbol{\lambda}}_{\boldsymbol{mn}}^{\boldsymbol{t}}=\frac{{\displaystyle {\sum}_{\boldsymbol{ij}=1}^{\boldsymbol{N}}}{\boldsymbol{D}}_{\boldsymbol{mn}}^{\boldsymbol{ij}}}{\boldsymbol{N}} $$

where *N* is the number of protein pairs containing a domain pair (*m, n*). The initial value of *λ*_*mn*_ was expressed as ${\sum}_{ij\kern0.5em =\kern0.5em 1}^N{P}_{mn}^{ij}/N$. We can estimate *λ*_*mn*_ by iterating between E- and M-steps to obtain the maximum likelihood estimation of *λ*_*mn*_. If ${\lambda}_{mn}^{t-1}$ was 0, then the non-zero *λ*_*mn*_ was updated in the EM algorithm; however, computational consumption was very high.

Although several domain pairs were found in the hundreds of protein pairs, only a few protein pairs were found to be interacting. As a result, an initial low *λ*_*mn*_ and high computational consumption in the E-step were obtained. To simplify computation, we assigned all of the initial low *λ*_*mn*_ to zero. In the case of large protein pair samples (*n* >30) containing a domain pair (*m*, *n*), if *λ*_*mn*_ fulfilled the following condition:

$$ \frac{{\boldsymbol{\lambda}}_{\boldsymbol{mn}}-\boldsymbol{p}}{\sqrt{\boldsymbol{p}*\left(1-\boldsymbol{p}\right)/\boldsymbol{n}}}<{\boldsymbol{Z}}_{0.05}=1.6448 $$

the value of *λ*_*mn*_ was set to zero and not used in the E-step. In the present study, *p = *0.00143 was the observed protein interaction probability and computed using the number of the experimentally confirmed PPIs to divide the total number of protein pairs across the six species. Therefore, we only considered *λ*_*mn*_ with initial values that were significantly higher than the observed protein interaction probability (*p*).

The EM algorithm was implemented in Matlab 2012b for two months by using the parallel computing toolbox. EM steps were repeated until the difference between two consecutive steps was <0.01 or the number of repeats >40. Estimates of *λ*_*mn*_ were then obtained and used for protein interaction prediction.

### FFT analysis of microarray dataset

FFT has been used to detect genes relevant to specific biological processes, such as cell cycle and cardian clock [20-22]. FFT converts a signal in the time domain into one in the frequency domain, thereby showing the magnitude of each frequency present in the signal. The formula is expressed as follows:

$$ {\boldsymbol{Y}}_{\boldsymbol{k}}=\frac{1}{\sqrt{\boldsymbol{N}}}{\displaystyle \sum_{\boldsymbol{n}=0}^{\boldsymbol{N}-1}}{\boldsymbol{X}}_{\boldsymbol{n}}{\boldsymbol{e}}^{-\boldsymbol{j}\boldsymbol{k}\left(\frac{2\boldsymbol{\pi}}{\boldsymbol{N}}\right)\boldsymbol{n}} $$

where *N* is the length of signal, and *k* is the frequency. Using this method, we can determine the frequency of a particular gene and its amplitude. The magnitude (*M*) at frequency *ω* = 1 of each gene was determined. The genes significantly correlated with cell cycle with magnitude *M* >0.5 at frequency *ω* = 1 were selected. To determine the peak expression time of each gene, we calculated phase value (*P*) by using the major frequency, and the peak expression time was computed using the following equation:

$$ {\boldsymbol{T}}_{\boldsymbol{peak}}=\left\{\begin{array}{c}\hfill \frac{\left(-\boldsymbol{P}\right)}{2\boldsymbol{\pi}}*48\kern3.25em  if\ P\  is\  negative\hfill \\ {}\hfill \frac{\left(-\boldsymbol{P}\right)}{2\boldsymbol{\pi}}*48+48\  if\ P\  is\  positive\hfill \end{array}\right. $$

With Fourier analysis, periodic genes within a genome can be identified, and the peak expression time of each gene can be determined.

We performed FFT analysis on time course microarrays in the asexual intraerythrocytic developmental cycle (IDC) of *P. falciparum* [23]. These expression profiles were obtained at intervals of 1 h for 48 h except on the 23rd and 29th h. The missing data were filled using the *k* nearest neighbor (KNN) method [24]. Before FFT analysis was performed, the expression value of each gene was centered by subtracting the mean value.

### Identification of membrane proteins

Proteins located in the membrane of human erythrocyte or malaria parasite should contain a transmembrane domain (TM) in their structures. They may or may not contain cleavable signal peptides in their sequences. To identify the human and malaria parasite membrane proteins that may interact, we used SignaIP and TMHMM to predict membrane proteins [25,26]. The sequence regions where predicted signal peptide and TM domain coexist were excluded because signal peptides are likely to be incorrectly predicted as TM domains by TMHMM. Proteins with multiple TM domains in their sequences were considered to be membrane proteins. The SignaIP program was run under default parameters except the D-cutoff value was adjusted to 0.33. The parameters for TMHMM were retained.

### Receiver operating characteristic (ROC) curve

The ROC curve representing the relationship between true positive rate (TPR) and FPR was used to evaluate the reliability of predicted protein interactions [27]. A protein pair was considered interacting if the interaction probabilities of the protein pairs were larger than the threshold value. TPR and FPR were defined as follows:

$$ \boldsymbol{T}\boldsymbol{P}\boldsymbol{R}=\frac{\boldsymbol{TP}}{\boldsymbol{TP}+\boldsymbol{F}\boldsymbol{N}},\kern0.5em \boldsymbol{F}\boldsymbol{P}\boldsymbol{R}=\frac{\boldsymbol{FP}}{\boldsymbol{FP}+\boldsymbol{T}\boldsymbol{N}} $$

Positive predictive values (PPV), also known as precision, were introduced to indicate the accuracy of prediction. PPV was computed as follows:

$$ \boldsymbol{P}\boldsymbol{P}\boldsymbol{V}=\frac{\boldsymbol{TP}}{\boldsymbol{TP}+\boldsymbol{F}\boldsymbol{P}} $$

If the predicted result showed high PPV, a high success rate could be obtained in experimental validation of predicted protein interactions.

### Gene ontology (GO) enrichment analysis

To understand the biological meaning of a large list of genes identified using the FFT method, we performed GO term enrichment analysis on these genes via the DAVID web server (http://david.abcc.ncifcrf.gov/) [28]. The GO terms of the gene molecular function were selected, and a gene set was considered to be enriched if the *p*-value was <0.05.

## Results

### Estimation of DDI probabilities using the EM algorithm

Protein domains are the structural units of proteins, and PPIs are mostly achieved through DDIs. Therefore, we first needed to estimate the DDI probabilities before inferring PPI probabilities. In our analysis, an EM algorithm was introduced to estimate the probability of each DDI.

**Figure 1:**
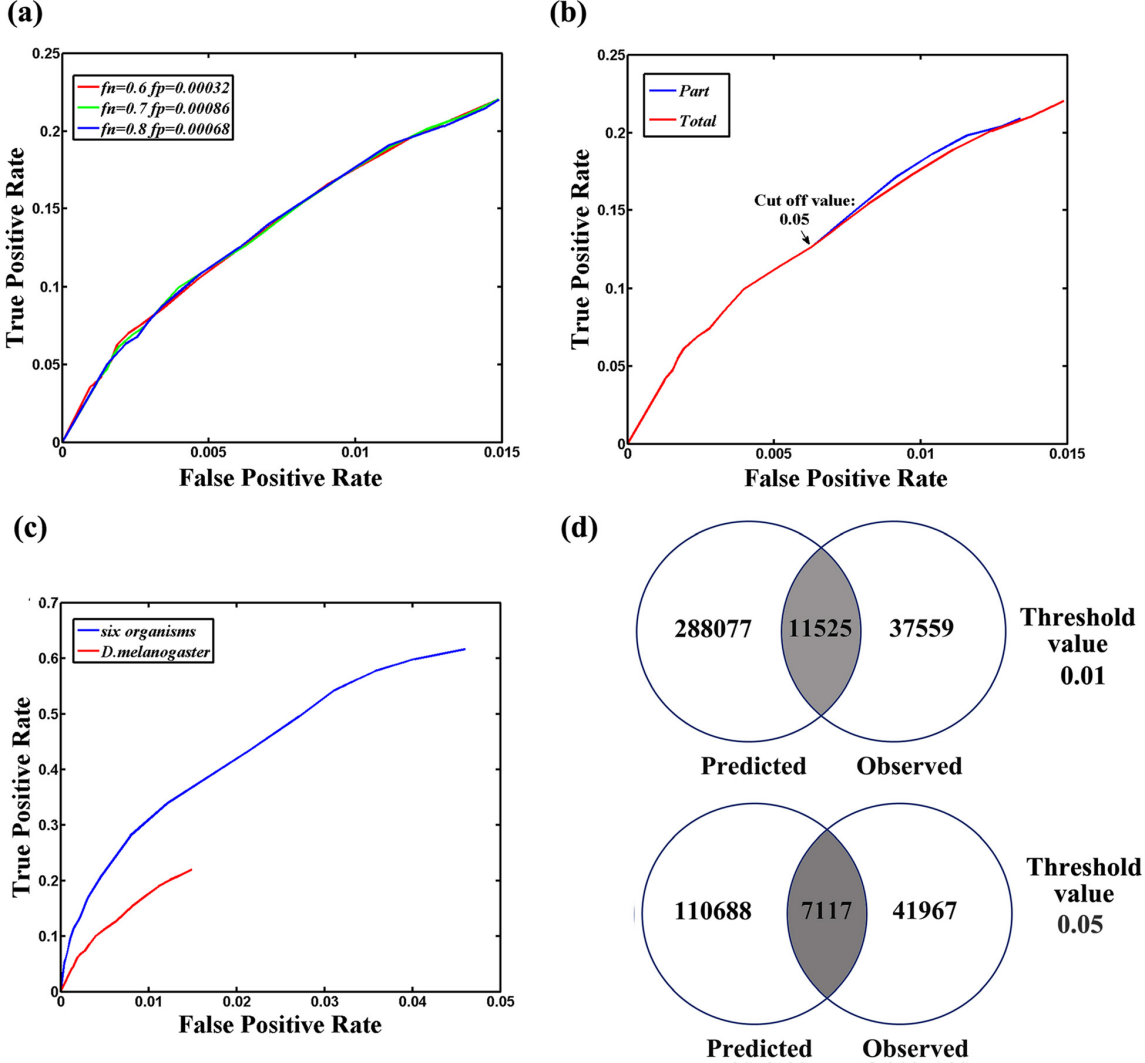
**Estimation of DDI probabilities using the EM algorithm. (a)** ROC curves of prediction results obtained using different *f*_*n*_ and *f*_*p*_ values. **(b)** ROC curves obtained based on total non-zero *λ*_*mn*_ and partly non-zero *λ*_*mn*_. **(c)** Comparison of ROC curves based on protein interactions from six species with that observed from information of *D. melanogaster* alone. **(d)** Venn diagram for number of predicted PPIs and observed PPIs in *S. cerevisiae*. Threshold value was set at 0.01 (upper panel) or 0.05 (lower panel).

This algorithm was initially applied to predict the DDI probabilities from the iPFAM and 3DID databases based on information pertaining to *D. melanogaster*. Considering that the protein interaction data contain several errors, we cannot determine these errors because actual protein interactions are unknown. In our analysis, a range of error rates were examined to assess the accuracy of this maximum likelihood method. Table 1 lists the *f*_*n*_ and *f*_*p*_ used in our analysis. As shown in Figure 1, the ROC curves exhibited no apparent difference when *f*_*n*_ and *f*_*p*_ were assigned to three different values, which suggested that prediction accuracy was robust with respect to *f*_*n*_ and *f*_*p*_. This finding is consistent with the results observed by Liu et al. [12].

**Table 1:** ***f***_***n***_ **and** ***f***_***p***_ **used in the EM algorithm.** ***t*** **represents assumed average number of interaction partners**

***t***	***f***_***n***_	***f***_***p***_
10	**0.7**	**0.00086**
15	**0.6**	**0.00032**
20	**0.8**	**0.00068**

Considering that the computational consumption of estimating all DDI probabilities was very high, we assigned the initial low *λ*_*mn*_ as zero (see Methods). The initial *λ*_*mn*_ significantly higher than the observed PPI probability (*p* = 0.00143) was used in EM algorithm. This simplification indicated that only parts of non-zero *λ*_*mn*_ were used for computation. To assess the effect of this alteration on the prediction accuracy of the EM algorithm, we fixed *f*_*n*_ and *f*_*p*_, and the ROC curves obtained using totally non-zero λ_*mn*_ were then compared with those obtained using partly non-zero λ_*mn*_. The ROC curves were nearly identical when the cut off value was set at ≥0.05 (Figure 1b). Slight differences were observed when the cut off value was <0.05. Similar trends were observed when *f*_*n*_ and *f*_*p*_ were assigned with different values (Additional file 2). Despite the slight differences in the ROC curves obtained using totally or partly non-zero λ_*mn*_, the time consumed by running the EM algorithm by using partly non-zero λ_*mn*_ was reduced by almost half.

Previous investigation had established that the prediction performance based on multiple organisms outperformed the performance obtained from only a single organism. Therefore, we integrated the protein interaction data from six organisms to estimate DDI probabilities. Our computer memory was limited; as such, the program could not be run using totally non-zero λ_*mn*_. Therefore, we treated λ_*mn*_ with a low value as zero and used the rest of the non-zero λ_*mn*_ to estimate DDI probabilities. Indeed, the prediction accuracy based on six organisms was higher than that based on the protein interaction data of a single organism. The maximum TPR level increased from 0.209 to 0.616 (Figure 1c). With the the same FPR value, the TPR value observed from the information of six organisms was apparently higher than that observed from *D. melanogaster* alone. On the basis of this result, we used the estimates of DDI probabilities to compute PPI probabilities.

Before estimating the interaction probabilities between human and parasite membrane proteins by using estimates of DDI probabilities, we accessed the performance of those DDI probabilities in inferring the PPIs of *S. cerevisiae*. The probability of each PPI was computed using the formula of *P*_*inter*_ (see Methods). At a threshold of 0.05, 117,805 protein pairs were predicted to be interacting; among these interactions, 7,117 protein interactions were identified (Figure 1d, upper panel). The TPR of this method was 0.145, and PPV reached 0.0604, which was nearly 42 times higher than the observed interaction probability (*p* = 0.00143). These results suggested that the PPIs inferred using the estimates of DDI probabilities were reliable. At a threshold of 0.01, 299,602 proteins were predicted to be interacting (Figure 1d, lower panel) and TPR and PPV were 0.235 and 0.0385, respectively. Although a decrease in the threshold value increased TPR, FPR was also found to be increased.

### Prediction of interactions between parasite and human membrane proteins

**Figure 2:**
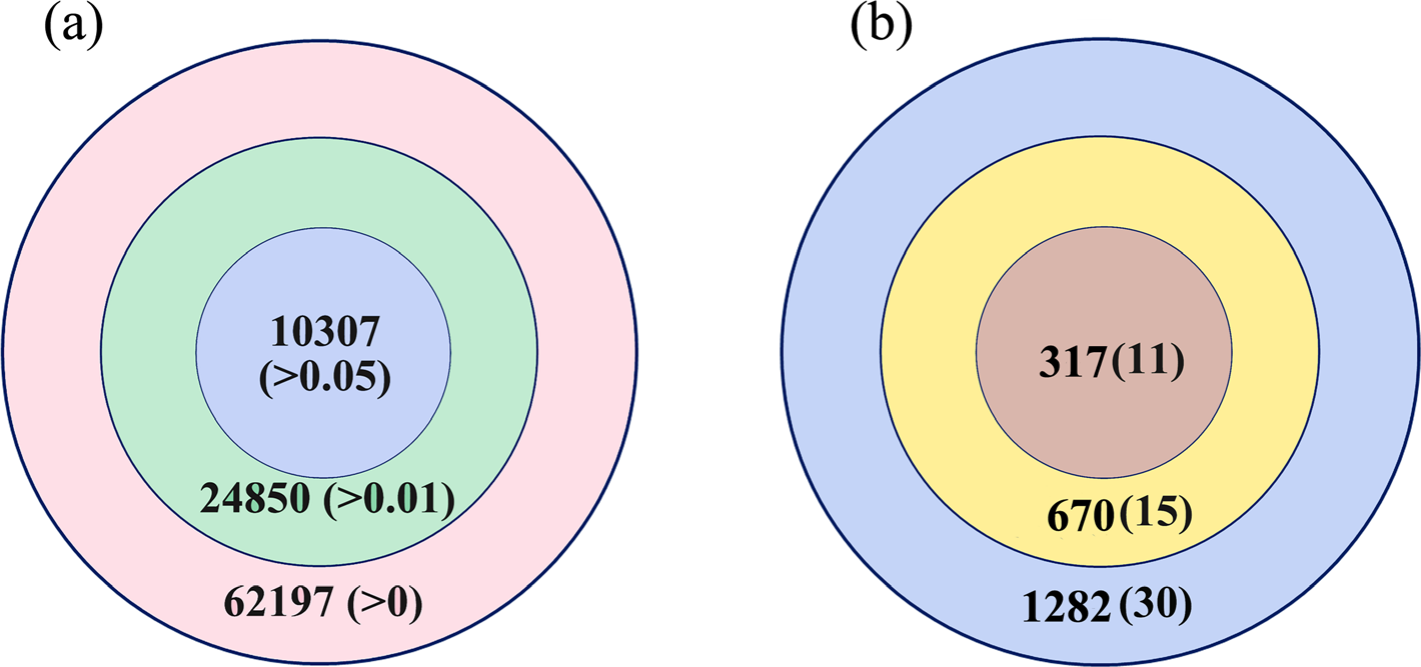
**Prediction of interactions between human and malaria parasite proteins. (a)** Venn diagram of the number of predicted membrane protein interactions when different threshold values were selected. Figures enclosed in parentheses represent the selected threshold values. **(b)** Evaluation of the performance of our method in predicting human and parasite protein interactions. Colors represent selected threshold values: blue for 0, yellow for 0.01, and brown for 0.05. Figures between parentheses represent number of interactions definitely predicted.

Interactions between parasite and human membrane proteins contribute to invasion of RBCs by the parasite. Therefore, such protein interactions should be studied to help illustrate the invasion process and provide new intervention avenues for controlling malaria. By combining the prediction results of SignaIP and TMHMM and filtering out proteins with no significant PDRs, we identified 3,787 and 490 membrane proteins in human and malaria parasite genome, respectively. To infer plausible interactions between membrane proteins, we used estimates of DDI probabilities and computed PPI probabilities. A total of 62,197 PPI probabilities were >0 (Figure 2a). At a threshold of 0.01, 24,850 protein pairs were predicted to be interacting. Notably, not all of the predicted interactions are involved in the invasion process, while some may be false positives.

Considering that significantly limited interactions were observed between human and parasite membrane proteins, we could not easily evaluate the prediction accuracy of our method. To demonstrate the viability of our method in predicting interactions between proteins from different species, 456 experimentally confirmed interactions between human and parasite proteins were used. After removing proteins not fulfilling the PDR conditions, we obtained a network consisting of 132 interactions between 66 parasite membrane proteins and 107 human membrane proteins. The observed interaction probability of this small-scale network was *p* = 0.0187. We then used this small-scale network to roughly assess the performance of our method in predicting interactions between human and parasite proteins. The interaction probabilities of these proteins were calculated using estimates of DDI probabilities. Figure 2b shows that 1,282 protein interaction probabilities were >0. The maximum TPR was 0.2270. At a threshold of 0.05, 11 of 317 protein interactions were predicted. TPR and PPV were 0.0833 and 0.0347, respectively, which were evidently lower than those obtained in *S. cerevisiae*. Even when the threshold was set at 0.01, only 15 interactions were predicted to be interacting. When TPR was increased to 0.114, PPV decreased to 0.0224, which was slightly higher than the observed interaction probability (*p* = 0.0187). The low TPR may be caused by several protein interactions in this network being false positives, since the observed interaction probability (*p* = 0.0187) was apparently larger than the interaction probability observed in the aforementioned six species (*p* = 0.00143). Low PPV suggests that integration of other information is necessary to improve our prediction accuracy.

We excluded falsely predicted PPIs by integrating the expression profiles of merozoite and human erythrocytes to improve the PPV of PPI prediction and to provide the direction for future experimental studies. The interactions between merozoite and human erythrocyte membrane proteins are mostly responsible for parasite invasion. Therefore, highly expressed proteins in merozoites and human erythrocytes should be identified to improve prediction accuracy.

### FFT identification of proteins highly expressed on merozoite membrane

**Figure 3:**
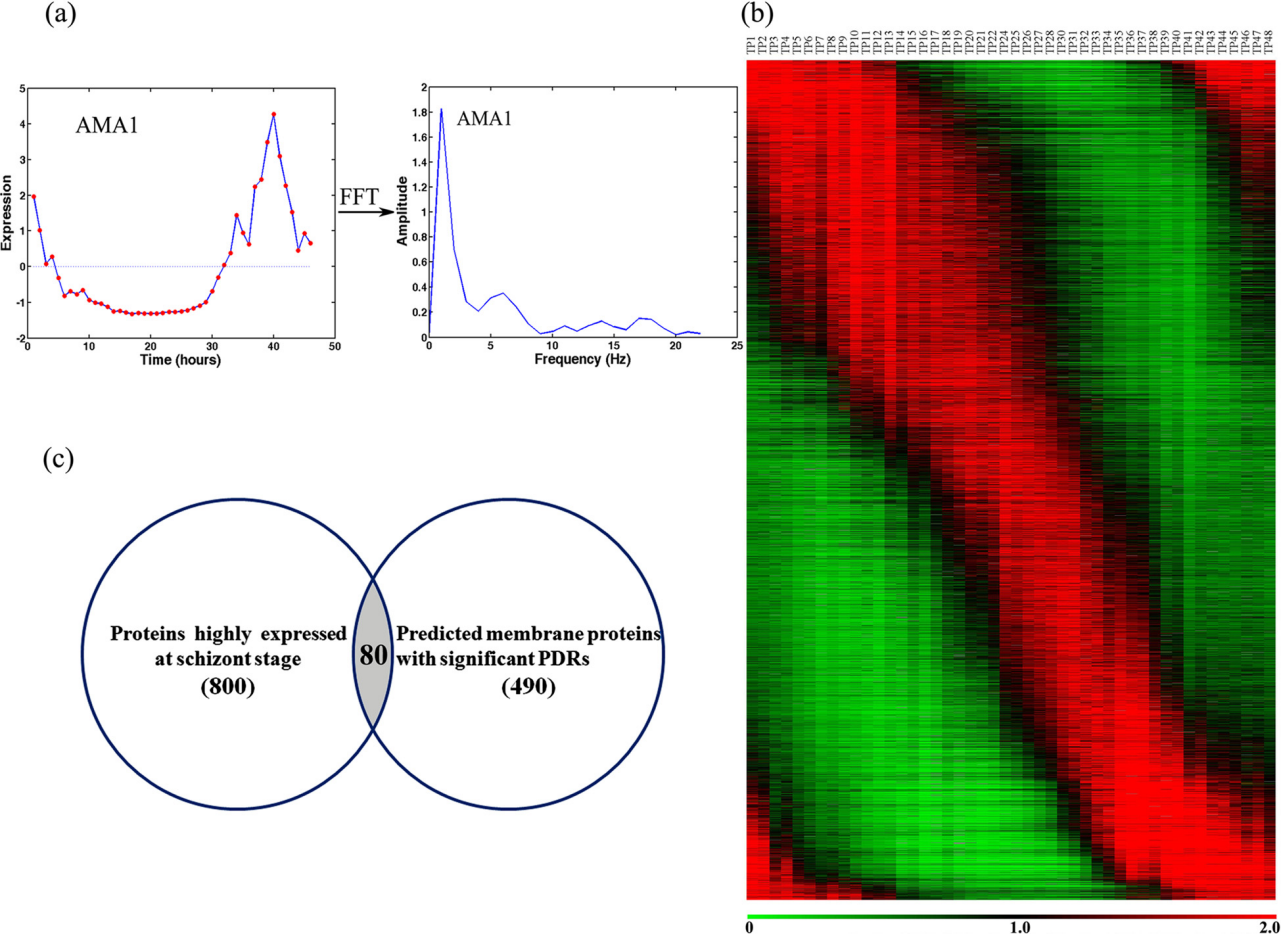
**Identification of malaria parasite genes highly expressed at schizont stage. (a)** PfAMA1 expression signal was converted from a time domain into a frequency domain by FFT. **(b)** Periodic genes identified using FFT were organized in increasing order of peak expression time. **(c)** Venn diagram for number of proteins found at malaria parasite schizont stage and that of proteins predicted to be located in the parasite membrane.

Considering that the IDC gene expression of *P. falciparum* was highly dynamic, we performed fast Fourier transform (FFT) analysis to extract the periodic genes that were highly expressed in the schizont stage. FFT is an approach used to compute a discrete Fourier transform of a finite length signal; this approach has also been applied for periodic genes whose expressions oscillate at one or more frequency. FFT converts an expression signal in a time domain into a frequency domain. Significant frequencies could be obtained by using this method. For example, the apical merozoite antigen (PfAMA1) plays a critical role in parasite invasion [29]. This gene was highly expressed in the schizont stage (Figure 3a, left panel). By conducting an FFT analysis, we obtained the amplitude of the AMA1 expression signal at each frequency (Figure 3a, right panel). Using this method, the expression profiles that are inherently noisy or lack differential expression can be filtered out to obtain the periodic genes.

To identify highly expressed genes in merozoites, we only need to extract the genes highly expressed in the schizont stage because mature schizonts consist of tens of merozoites. We performed FFT analysis on a published microarray dataset [23]. Probes whose spectral amplitudes were >0.5 at *ω* = 1 were considered to be significant periodic probes. The peak expression time of each periodic gene was calculated using a previously described method (T_*peak*_ calculation, see Methods). The profiles of the periodic genes were organized by increasing the time of peak expression (Figure 3b). The results of Giemsa staining of the major morphological stages throughout the IDC had indicated that schizonts initially appeared at 25 h after the erythrocytes were invaded. In our study, genes with peak expression times at intervals of 30 h and 48 h were considered highly expressed in merozoites. A total of 3,442 genes contained significant frequency components, of which 800 were highly expressed in the schizont stage.

Among these 800 genes, approximately 330 genes were annotated with molecular functions. The results of enrichment analysis indicated that the genes implicated in protein serine/threonine kinase activity were significantly enriched (Table 2). This result is consistent with a previous report that the invasion of erythrocytes is sensitive to kinase inhibitors [30]. Genes related to calcium binding activity, cytoskeletal protein binding, and oxidoreductase activity were also significantly enriched. This result is also similar to that reported by Bozdech et al. [23]. The functions of the 470 remaining genes await further investigation.

**Table 2:** **GO term enrichment analysis of genes highly expressed at schizont stage**

**Term**	**Count**	**Percentage**	***P*** **-Value**
GO:0004674 protein serine/threonine kinase activity	**25**	**3.156566**	**2.26E-05**
GO:0004672 protein kinase activity	**27**	**3.409091**	**1.38E-04**
GO:0016773 phosphotransferase activity, alcohol group as acceptor	**31**	**3.914141**	**8.50E-04**
GO:0016301 kinase activity	**35**	**4.419192**	**0.002383353**
GO:0005509 calcium ion binding	**12**	**1.515152**	**0.002680592**
GO:0003779 actin binding	**8**	**1.010101**	**0.005397112**
GO:0008092 cytoskeletal protein binding	**8**	**1.010101**	**0.01217268**
GO:0016772 transferase activity, transferring phosphorus-containing groups	**44**	**5.555556**	**0.013433805**
GO:0016491 oxidoreductase activity	**22**	**2.777778**	**0.01710942**
GO:0004197 cysteine-type endopeptidase activity	**8**	**1.010101**	**0.017219233**
GO:0008234 cysteine-type peptidase activity	**9**	**1.136364**	**0.022687224**
GO:0004252 serine-type endopeptidase activity	**5**	**0.631313**	**0.027441466**
GO:0042578 phosphoric ester hydrolase activity	**11**	**1.388889**	**0.037112884**
GO:0005200 structural constituent of cytoskeleton	**5**	**0.631313**	**0.043219537**

To infer interactions between merozoite and human erythrocyte proteins, we focused on membrane proteins of these 800 proteins expressed highly in the schizont stage. We overlapped these 800 proteins and 490 parasite membrane proteins previously identified and found that 80 membrane proteins were highly expressed in the schizont stage (Figure 3c). To identify membrane proteins of RBCs, we used published mass spectral datasets [31,32]. A total of 2,396 proteins were found to be expressed in RBCs; among them, 221 were predicted to be membrane proteins and had significant PDRs.

### Prediction of protein interactions involved in malaria parasite invasion of human erythrocytes

On the basis of previously computed 62,197 probabilities between human and parasite membrane proteins, we obtained a network consisting of 80 parasite proteins and 221 human proteins after the proteins not expressed in the schizont stage and those not found in RBCs were filtered out. A total of 960 interactions with probabilities >0 were found. At a threshold of 0.01, 467 interactions were predicted. Considering that experimentally discovered surface protein interactions are limited, we could not evaluate the accuracy of our prediction. Six non-interacting protein pairs were manually collected from the third high confidence dataset. These non-interacting protein pairs consisted of two parasite membrane proteins and three human membrane proteins. Our prediction results indicated that all of the probabilities of these protein pairs were 0, suggesting that no interactions were found between those proteins. Thus, our predicted results are consistent with the experimental results. Further experimental validation is needed to fully assess the accuracy of our predictions.

**Figure 4:**
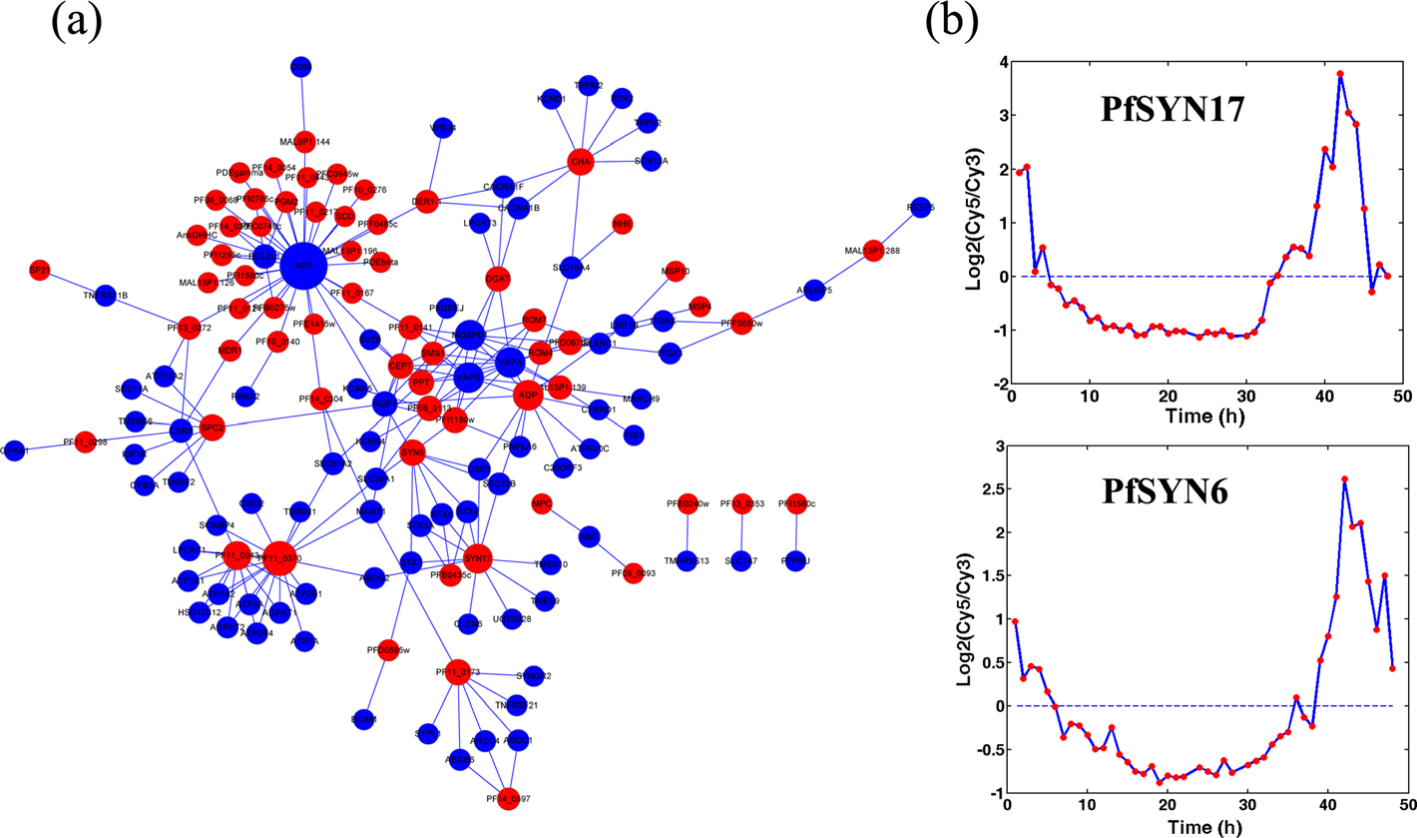
**Predicted PPIs related to parasite invasion. (a)** Predicted network consisted of 205 interactions. Network was visualized using Cytoscape [35]. Red nodes of this network represent *P. falciparum* proteins, and blue nodes represent human proteins. Node size was scaled to node degree such that larger node size indicates higher node degree. The text in each node represents gene symbol or gene name. **(b)** mRNA expression of SNAREs (PfSYN17 and PfSYN6) from a published microarray dataset. The peak expression timepoints of these genes were 42 h.

To increase the PPV of prediction and provide directions for future experimental studies, we set the threshold at 0.05 and built a small network containing 205 interactions (Figure 4a and Additional file 3: Table S2). Considering that hub proteins are the principal agents in a PPI network and affect network function and stability, we focused on proteins with a large number of interactions in this small-scale network. APP exhibited the highest degree of interactions, indicating that proteins in malarial parasites possibly interact with this membrane protein and eventually invade RBCs. APP is a cell surface receptor involved in Alzheimer’s disease and cerebral amyloid angiopathy [33,34]. Whether this gene product participates in the parasitic invasion of erythrocytes should be further validated experimentally. The rest of the human proteins predicted to be related to the invasion process were VAPA, VAPB, and MOSPD1 (Table 3a). Among these three proteins, VAPA and VAPB are VAMP associated proteins and are involved in vesicle trafficking, exocytosis, and endocytosis.

**Table 3:** **Proteins with node degrees higher than six in the predicted network**

**a.** Human proteins with node degrees higher than six.	
Gene symbols	Description
APP	**amyloid beta (A4) precursor protein**
VAPA	**VAMP (vesicle-associated membrane protein)-associated protein A**
VAPB	**VAMP (vesicle-associated membrane protein)-associated protein B and C**
MOSPD1	**motile sperm domain containing 1**
b. Parasite proteins with node degrees higher than six.	
Gene symbols	Description
PF11_0370	**3-oxo-5-alpha-steroid 4-dehydrogenase**
AQP	**aquaglyceroporin**
SYN17	**syntaxin, Qa-SNARE family**
PF11_0343	**LEM3/CDC50 family protein**
CHA	**Mitochondrial calcium-proton antiporter**
SYN6	**SNARE protein**
SPC2	**signal peptidase complex subunit 2**
PF11_0173	**oligosaccharyl transferase STT3 subunit**

Eight parasite proteins in this network exhibited degrees of interaction more than six and were selected for further analysis (Table 3b). Some of these proteins, including PFF0170w, PFC0912w, and PF11_0173, were false positives because they are not localized on the plasma membrane of parasites and hence cannot interact with human proteins. The parasite SNARE proteins PfSyn17 and PfSyn6 were predicted to interact with human proteins. The peak expression times of these two proteins based on the microarray data were at 42 h (Figure 4b). Considering that SNARE proteins mainly regulate vesicle exocytosis and are involved in protein secretion [36,37], we speculate that the inferred interaction between SNARE proteins and human erythrocyte proteins may stimulate protein secretion by microneme and rhoptry. This may in turn facilitate the ability of malaria parasite to invade erythrocytes. Thus, our results suggest that SNARE proteins may possibly be involved in parasite invasion.

On the basis of these network analysis results, we chose parasite SNARE proteins and human APPs for further biological experimental studies.

## Discussion

In the present study, we sought to elucidate the molecular basis of invasion of RBCs by malarial parasites. We used the EM algorithm to estimate the DDI probabilities based on currently available large-scale protein interaction datasets, and then used the estimates of DDI probabilities to predict interactions between human RBCs and merozoite membrane proteins. We also integrated gene expression information to reduce false positives and thus improve our prediction accuracy. Using FFT, we identified the genes highly expressed in the schizont stage. By combining estimated PPI probabilities and gene expression profiles, we predicted a network consisting of 205 PPIs between parasite and human membrane proteins. The results of further analysis suggested that SNARE proteins in parasites and APP in humans may play an important role in the invasion of erythrocytes.

Although we inferred possible interactions between human and malaria parasite proteins, our prediction did not capture several discovered protein interactions related to parasite invasion of RBCs because we only considered possible interactions between proteins that satisfy the PDR condition. For instance, previous experimental investigations reported that several interactions between parasite proteins and human proteins, including MSP1-Band3 and EBA175-Glycophorin A [38,39], are involved in invasion. Our predicted interactions did not include these two interactions because the parasite proteins MSP1and EBA175 do not contain any domain found in the six species and were therefore not used for PPI probability estimation. Actually, a total of 815 parasite proteins that may be located in the parasite membrane were identified. However, only 490 of those contain domains that are conserved in all six species. This finding indicated that our method could not be used to predict whether the 325 remaining proteins interact with human membrane proteins. Furthermore, a total of 839 proteins were identified from the merozoite proteome [40], of which 62 were assumed to be surface proteins. Only 22 surface proteins were found to share recognizable domains across the six organisms, which implied that interactions mediated by the remaining 40 proteins could not be inferred from our study. Expansion of the PPI collection could overcome this limitation. Another limitation of our method is that, since we only considered the information of domain-domain interactions, PPIs mediated by posttranslational modifications, such as those in GYPC-EBA140 and GYPB-EBL1 [41,42], cannot be inferred from our results. To our knowledge, there is no computational method suitable for inferring PPIs mediated by posttranslational modifications.

In addition, interactions mediated by secretory or peri-membrane proteins are also not predicted because they were excluded from our analysis for the reason that most secretory parasite proteins contain domains not found in all six organisms and identification of human peri-membrane proteins is not feasible. In our study, we only predicted interactions between membrane proteins in parasites and human RBCs. However, several secretory parasite proteins and human perimembrane proteins are known to be utilized by parasites in the invasion of RBCs. For instance, PfRh5 proteins secreted by parasite rhoptry are involved in parasite invasion of erythrocytes by directly interacting with the surface protein Basigin in RBCs [43]. The human perimembrane protein SEMA7A interacts with parasite membrane proteins PfMtrap and participates in parasite invasion [44]. Therefore, extending the protein candidates to human perimembrane and parasite secretory proteins is the best solution to capture interactions in which these kinds of proteins are involved.

We found that the results of incorporating information from six species outperformed those obtained from *D. melanogaster* alone, which allowed us to make more informative inferences regarding protein interactions. A network composed of interactions between human and parasite proteins was then inferred. Assessment of our predictions using a small-scale protein interaction dataset indicated that our prediction accuracy was lower than that obtained in *S. cerevisiae*. This phenomenon may be caused by several protein interactions being false positives in the small-scale dataset, and suggested that other information, such as gene expression profile and gene ontology information, should be integrated to improve prediction accuracy [45]. In our analysis, gene expression profile data was incorporated to increase the prediction accuracy. In addition, GO information, particularly information related to protein localization, could be integrated to further improve the accuracy of our predictions. For example, in our predicted PPI network, several proteins were found to be located on the membrane of mitochondria and the endoplasmic reticulum. This result suggested that proteins located on the membrane of these two cellular components may be falsely predicted to interact with human membrane proteins. The exclusion of interactions in which such proteins participate possibly increased our prediction accuracy. Another key factor affecting prediction performance is the selection of threshold value. Predicted interactions depend on a chosen threshold value. Although an increase in the threshold value likely improves prediction accuracy, the TPR is evidently reduced. Considering the incompleteness of domain information and the unknown number of definitely interacting protein pairs, we could not easily set a threshold value to determine whether or not a protein pair exhibits interaction.

Despite these limitations, our predicted interactions revealed that SNARE proteins that participate in protein secretion may be involved in malaria parasite invasion of erythrocytes. This result is consistent with previous reports that protein secretion via microneme and rhoptry are necessary to induce parasite invasion of RBCs [1]. Predicted interactions also indicated that human APP may function in parasite invasion. The erythrocyte proteome data shows that APP is expressed at a medium level in RBCs [32]. Analysis of dataset from the 1000 genome database [46] indicated that several SNPs of APP, such as rs216772, rs2829992 and rs216773, are more prevalent in among Africans. Understanding the significance of the relation between these SNPs and parasite invasion requires further investigation. Considering that the functions of APP and SNARE in parasite invasion have not been elucidated previously, we will conduct a series of biological experiments to study them. Although experimental validation of these interactions was hampered by difficulty in expression of parasite proteins in mammalian cells, some encouraging progress has been made by malariaologists [47,48].

## Conclusions

In the present study, the EM algorithm was used to estimate probabilities of DDIs, which were then applied to infer possible protein interactions between human RBCs and parasite merozoite membrane proteins. Gene expression data was integrated to filter out false positives. FFT was introduced to identify genes expressed highly in the schizont stage, while published data was utilized to search for membrane proteins of RBCs. A network consisting of 205 PPIs, including several novel interactions, was obtained. Our results help elucidate the molecular mechanism of parasite invasion and provide promising research directions for further experimental studies.

## Additional files

Additional file 1: Table S1. Protein interaction datasets used for DDI probability estimation. Additional file 2: Figure S1. Comparison of ROC curves obtained from totally non-zero *λ*_*mn*_ with those based on partly non-zero *λ*_*mn*_ after *f*_*n*_ and *f*_*p*_ were specified. (a) ROC curves observed when *f*_*n*_ = 0.6 and *f*_*p*_ = 0.00032. (b) ROC curves observed when *f*_*n*_ = 0.8 and *f*_*p*_ = 0.00068. Additional file 3: Table S2. Predicted protein interactions between parasite and erythrocyte membrane proteins.
