# Supplementary material for: Computational prediction of protein interactions related to the invasion of erythrocytes by malarial parasites
Source: BMC Bioinformatics. 2014 Nov 30;15(1):393. doi: 10.1186/s12859-014-0393-z (PMC4265449; doi:10.1186/s12859-014-0393-z)
Supplement: Additional file 1: Table S1. — Protein interaction datasets used for DDI probability estimation. [file 12859_2014_393_MOESM1_ESM.docx]

| Species | *A. thaliana* | *C. elegans* | *D. melanogaster* | *H.sapiens* | *M. musculus* | *S. pombe* |
| --- | --- | --- | --- | --- | --- | --- |
| Number of interactions | **9478** | **3052** | **21752** | **94396** | **7409** | **3828** |
| Number of proteins | **4502** | **1974** | **5583** | **11184** | **3527** | **1610** |
