# Supplementary figures and images for: Computational prediction of protein interactions related to the invasion of erythrocytes by malarial parasites
Source: BMC Bioinformatics. 2014 Nov 30;15(1):393. doi: 10.1186/s12859-014-0393-z (PMC4265449; doi:10.1186/s12859-014-0393-z)

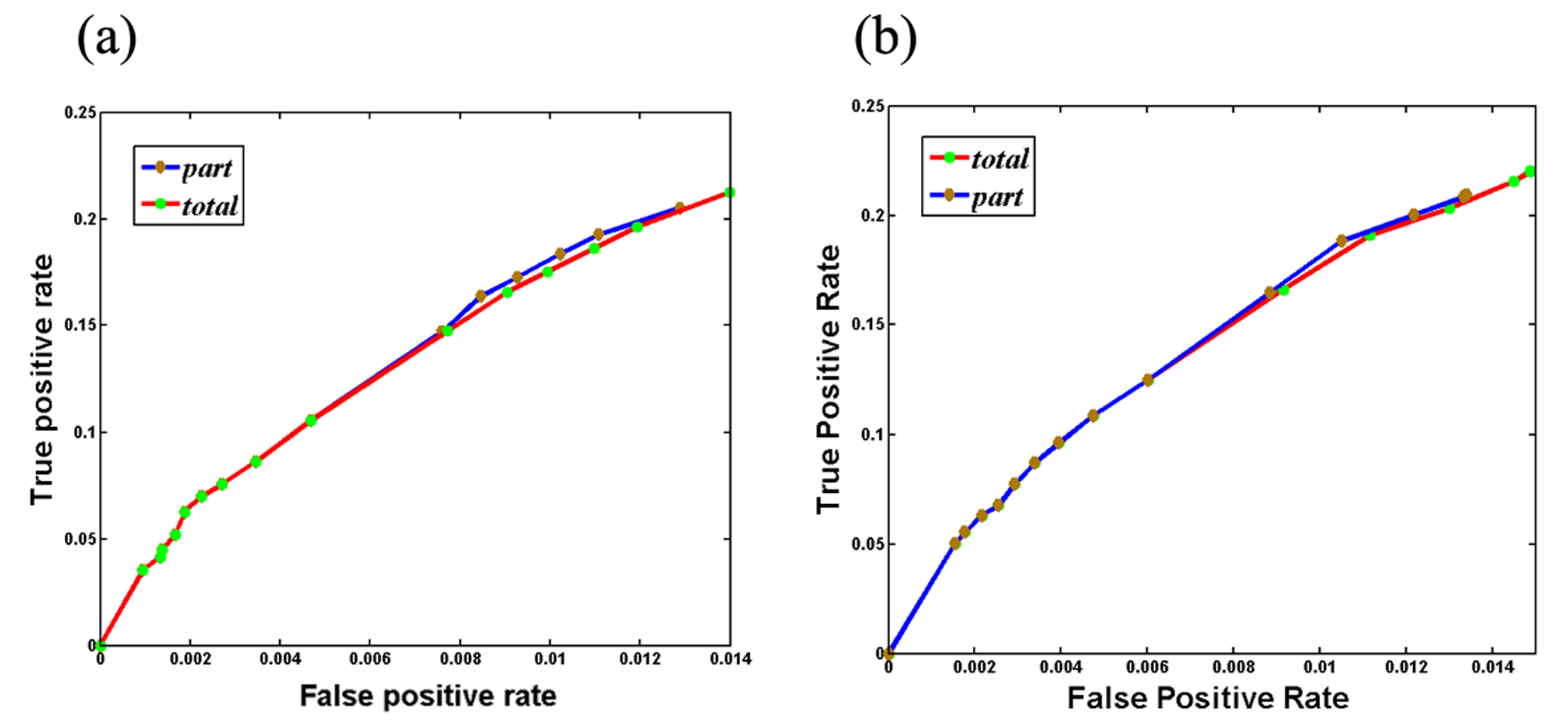

Supplement: Additional file 2: Figure S1. — Comparison of ROC curves obtained from totally non-zero λ mn with those based on partly non-zero λ mn after f n and f p were specified. (a) ROC curves observed when f n = 0.6 and f p = 0.00032. (b) ROC curves observed when f n = 0.8 and f p = 0.00068. [file 12859_2014_393_MOESM2_ESM.tiff]
