# Supplementary material for: Computational prediction of protein interactions related to the invasion of erythrocytes by malarial parasites
Source: BMC Bioinformatics. 2014 Nov 30;15(1):393. doi: 10.1186/s12859-014-0393-z (PMC4265449; doi:10.1186/s12859-014-0393-z)
Supplement: Additional file 3: Table S2. — Predicted protein interactions between parasite and erythrocyte membrane proteins. [file 12859_2014_393_MOESM3_ESM.docx]

| P.falciparum membrane proteins | Human membrane proteins |
| --- | --- |
| PF11_0173 | ABCB6 |
| PF14_0597 | ABCB6 |
| PFI1560c | PTPRU |
| ROM4 | PLXNC1 |
| ROM7 | PLXNC1 |
| DGAT | LPCAT3 |
| PF11_0173 | ABCC4 |
| PF14_0597 | ABCC4 |
| PPT | PKDREJ |
| PFF0650w | ARL6IP5 |
| MAL13P1.288 | ARL6IP5 |
| PF11_0343 | AGPAT1 |
| PF11_0370 | AGPAT1 |
| PF11_0343 | AGPAT2 |
| PF11_0370 | AGPAT2 |
| AQP | PNPLA6 |
| PFI1180w | PNPLA6 |
| SYN17 | TMED10 |
| PF11_0370 | SCAMP4 |
| SYN17 | CLCN6 |
| CHA | SLC15A4 |
| AQP | SLC15A4 |
| NHE | SLC15A4 |
| SPC2 | TMEM56 |
| SPC2 | CYB5A |
| PF13_0272 | ATP13A2 |
| SPC2 | ATP13A2 |
| SPC2 | SEC11A |
| PF08_0093 | NNT |
| MPC | NNT |
| PF11_0173 | TNFRSF21 |
| SPC2 | IGF1R |
| PF08_0068 | APP |
| MAL8P1.144 | APP |
| CEPT | APP |
| PFF0485c | APP |
| PF10_0140 | APP |
| PF10_0276 | APP |
| PF11_0124 | APP |
| PF11_0141 | APP |
| PF11_0167 | APP |
| PF11_0217 | APP |
| PF11_0443 | APP |
| PF14_0054 | APP |
| PF14_0260 | APP |
| PF14_0304 | APP |
| DER1-1 | APP |
| PGM2 | APP |
| PFB0275w | APP |
| SCD | APP |
| MDR1 | APP |
| PFE1415w | APP |
| SYN6 | APP |
| PFI0785c | APP |
| PFI1295c | APP |
| PFI1580c | APP |
| PDEbeta | APP |
| PDEgamma | APP |
| MAL13P1.126 | APP |
| MAL13P1.196 | APP |
| PF13_0272 | APP |
| AnkDHHC | APP |
| PFC0740c | APP |
| PFC0945w | APP |
| CEPT | AQP1 |
| SMS1 | AQP1 |
| PF11_0141 | AQP1 |
| AQP | AQP1 |
| PFI1180w | AQP1 |
| SPC2 | AQP1 |
| PFF0650w | ITGA3 |
| PFD0675w | ITGA3 |
| PFF0650w | ITGAX |
| PFD0675w | ITGAX |
| CHA | KCND1 |
| PF08_0113 | KCNN4 |
| PFD0595w | BCAM |
| MAL8P1.144 | CD99 |
| PF11_0173 | ABCC1 |
| PF14_0597 | ABCC1 |
| PF11_0343 | ATP1A1 |
| PF11_0370 | ATP1A1 |
| PF11_0343 | ATP1A2 |
| PF11_0370 | ATP1A2 |
| PF11_0343 | ATP2B1 |
| PF11_0370 | ATP2B1 |
| PF11_0343 | ATP2B4 |
| PF11_0370 | ATP2B4 |
| PF11_0370 | CISD2 |
| PF11_0343 | ATP4A |
| PF11_0370 | ATP4A |
| MAL13P1.139 | FIS1 |
| PF11_0343 | HSD17B12 |
| PF11_0370 | HSD17B12 |
| CHA | TRPV2 |
| AQP | ATP6V0C |
| MSP10 | LRP1B |
| AQP | LRP1B |
| MSP4 | LRP1B |
| PF11_0370 | ATP7A |
| SYN17 | UGT2B28 |
| SYN17 | TMED9 |
| PF11_0370 | SEC61A2 |
| PF14_0304 | SEC61A2 |
| PPT | SEC61A2 |
| AQP | USE1 |
| SYN17 | USE1 |
| SYN6 | USE1 |
| CEPT | MOSPD1 |
| SMS1 | MOSPD1 |
| PF08_0113 | MOSPD1 |
| PF11_0141 | MOSPD1 |
| AQP | MOSPD1 |
| PFD0675w | MOSPD1 |
| ROM4 | MOSPD1 |
| PPT | MOSPD1 |
| PFI1180w | MOSPD1 |
| MAL13P1.139 | MOSPD1 |
| ROM7 | MOSPD1 |
| DGAT | MOSPD1 |
| PF10_0140 | PANX2 |
| AQP | C20ORF3 |
| PF14_0260 | BCL2L1 |
| PGM2 | BCL2L1 |
| PFB0275w | BCL2L1 |
| PFI0785c | BCL2L1 |
| PFI1295c | BCL2L1 |
| CHA | RYR2 |
| CHA | SCN10A |
| PF11_0370 | TMBIM1 |
| PF13_0353 | SLC1A7 |
| PFB0435c | STX1A |
| SYN17 | STX1A |
| SYN6 | STX1A |
| PFB0435c | STX4 |
| SYN17 | STX4 |
| SYN6 | STX4 |
| PFB0435c | STX5 |
| SYN17 | STX5 |
| SYN6 | STX5 |
| PF11_0173 | SYPL1 |
| SP21 | TNFRSF1B |
| PF13_0272 | TNFRSF1B |
| CHA | TRPM2 |
| CHA | CACNA1B |
| DER1-1 | CACNA1B |
| DGAT | CACNA1B |
| PF08_0113 | SLC30A1 |
| PF11_0370 | SLC30A1 |
| PFI1180w | SLC30A1 |
| CHA | CACNA1F |
| DER1-1 | CACNA1F |
| DGAT | CACNA1F |
| DER1-1 | VPS24 |
| MAL13P1.288 | REEP5 |
| PF11_0343 | LPCAT1 |
| PF11_0370 | LPCAT1 |
| AQP | CYBRD1 |
| PF11_0298 | CANX |
| PF11_0343 | CANX |
| MDR1 | CANX |
| PF13_0272 | CANX |
| PFE0240w | TMPRSS13 |
| CEPT | MAGT1 |
| PF11_0173 | MAGT1 |
| PF11_0370 | MAGT1 |
| PF14_0304 | MAGT1 |
| PFD0595w | STX7 |
| SYN17 | STX7 |
| SYN6 | STX7 |
| PF08_0113 | KCNK5 |
| PF11_0298 | GPAA1 |
| PF11_0173 | SYNGR2 |
| CEPT | VAPB |
| SMS1 | VAPB |
| PF08_0113 | VAPB |
| PF11_0141 | VAPB |
| AQP | VAPB |
| PFD0675w | VAPB |
| ROM4 | VAPB |
| PPT | VAPB |
| PFI1180w | VAPB |
| MAL13P1.139 | VAPB |
| ROM7 | VAPB |
| DGAT | VAPB |
| CEPT | VAPA |
| SMS1 | VAPA |
| PF08_0113 | VAPA |
| PF11_0141 | VAPA |
| AQP | VAPA |
| PFD0675w | VAPA |
| ROM4 | VAPA |
| PPT | VAPA |
| PFI1180w | VAPA |
| MAL13P1.139 | VAPA |
| ROM7 | VAPA |
| DGAT | VAPA |
| MAL13P1.139 | MARCH9 |
| SPC2 | TM9SF2 |
| PF11_0370 | ABCG2 |
| SYN17 | ABCG2 |
| AQP | SEC22B |
| SYN17 | SEC22B |
| SYN6 | SEC22B |
| PPT | SV2B |
